# Whole genome sequences of *Lactococcus lactis* KCKM 0851 isolated from green onion kimchi

**DOI:** 10.1128/mra.00994-23

**Published:** 2023-12-08

**Authors:** Hye In Ko, So-Rim Kim, Chae-Rim Jeong, Mo Eun Lee, Jong-Bang Eun, Tae-Woon Kim

**Affiliations:** 1 Technology Innovation Research Division, World Institute of Kimchi, Gwangju, South Korea; 2 Department of Integrative Food, Bioscience and Biotechnology, Chonnam National University, Gwangju, South Korea; Rochester Institute of Technology, Rochester, New York, USA

**Keywords:** *Lactococcus lactis*, lactic acid bacteria, whole genome, kimchi, KCKM, KACC, probiotics

## Abstract

*Lactococcus lactis* KCKM 0851 isolated from green onion kimchi is a probiotic candidate and can be used as a starter culture for kimchi and dairy products. The whole-genome data of this strain will help us understand its genetics and metabolic characteristics.

## ANNOUNCEMENT

*Lactococcus lactis* is a Gram-positive bacterium primarily isolated from fermented milk and occasionally from fermented vegetables, including kimchi (1). During dairy production, *L. lactis* metabolizes lactose into lactic acid and converts proteins into flavor compounds, thereby contributing to the taste (1). *L. lactis* strains isolated from plant-based products perform different metabolic actions, and when applied to dairy products, they can produce products with new flavors (2). In this study, we performed whole-genome sequencing of *L. lactis* KCKM 0851 (KACC 23088) isolated from a plant-based product, green onion kimchi, which will aid in understanding its distinct metabolic activities and provide foundational information for its application as a starter culture.

First, green onion kimchi was finely ground and filtered through gauze. The extracted kimchi juice was diluted with sterile 0.85% NaCl solution, spread on MRS agar, and incubated at 30°C for 48 hours under anaerobic conditions. Next, single colonies were isolated and subcultured three times. The 16S rRNA gene sequencing of the isolates was performed as reported (3) and confirmed using EzBioCloud (https://www.ezbiocloud.net/identify). The total genomic DNA of *L. lactis* KCKM 0851 cultured on MRS agar was extracted for library preparation using the Maxwell Prokaryote SEV DNA Purification Kit (Promega, Madison, WI, USA), following manufacturer’s instructions. The DNA was subsequently sheared using Megaruptor 3 (Diagenode, Denville, NJ, USA) and purified with AMPure PB (Pacific Biosciences, Menlo Park, CA, USA). The library was prepared using PacBio SMRTbell prep kit 3.0. and sequenced on a Sequel II system (Pacific Biosciences) by Macrogen (Seoul, Republic of Korea). Total 63,052 HiFi reads were obtained with a total mean length of 8,817 bp and assembled with SMRTlink v11.0.0.146107 using the Hierarchical Genome Assembly Process (Genome Length: 5 M, Downsampled coverage: 20, Phred Scale: 30) (4). For error correction, the library was prepared using the TruSeq Nano DNA High Throughput Library Prep Kit (Illumina, San Diego, CA, USA). Sequencing was conducted on a NovaSeq6000 platform (Illumina) in a paired-end (2 × 150 bp) mode, producing 22,470,840 raw reads. In total, 15,395,642 filtered reads were produced using in-house script. Illumina reads were trimmed using Trimmomatic 0.38 (ILLUMINACLIP:Adapter.fasta:2:30:10:8:true LEADING:15 TRAILING:15 SLIDINGWINDOW:4:15 MINLEN:36) (5), and the trimmed reads were used three times to correct the assembly using Pilon v1.21 (--fix bases,gaps,local --nostrays --mindepth 0.01) (6). The assembly quality was assessed using BUSCO v. 5.1.3 (-m genome) (7).

The sequenced *L. lactic* KCKM 0851 genome comprised one chromosome and four contigs with a combined length of 2,526,315 bp, N_50_ value of 2,437,676, GC content of 35.0%, and depth of 206.1× (Table 1). Genome annotation was conducted using Prokka v1.14.6 (--rnammer --addgenes --gcode 11) (8), with further annotations using InterProScan v5.30–69.0 (-goterms) (9) and PSI-Blast v2.4.0 (-evalue 1.0E-5 -num_descriptions 5 -num_alignments 5) (10), in conjunction with EggNOG DB v4.5 (11). Finally, 2,505 CDSs, 61 tRNAs, and 19 rRNAs were identified. Additionally, secondary metabolite gene clusters were analyzed using antiSMASH leading to the detection of RaS-RiPP, T3PKS, three types of RiPP-like, and Betalactone.

**TABLE 1:** Summary of the assembled whole-genome sequence of *L. lactis* KCKM 0851

	Contig 1	Contig 2	Contig 3	Contig 4	Contig 5	Entire genome
Alias	Chromosome	Contig	Contig	Contig	Contig	
Length (bases)	2,437,676	45,154	24,049	12,584	6,852	2,526,315
GC (%)	35.1	33.0	33.8	34.1	32.7	35.0
Depth	194.1	792.8	329.0	154.5	273.0	206.1
CDS	2,417	44	27	8	9	2,505
tRNA	61	0	0	0	0	61
rRNA	19	0	0	0	0	19
Circular	Yes	Yes	Yes	Yes	Yes	
Complete and single-copy BUSCOs (S)						121
Complete and duplicated BUSCOs (D)						1
Fragmented BUSCOs (F)						2
Missing BUSCOs (M)						0
Total BUSCO groups searched						124

## Data Availability

For *L. lactis* KCKM 0851, the respective whole-genome sequence, BioProject, BioSample, and SRA accession numbers are JAVKVG000000000, PRJNA1012351, SAMN37256208, and SRR26415136.
